# Supplementary material for: Mechanisms governing the pioneering and redistribution capabilities of the non-classical pioneer PU.1
Source: Nat Commun. 2020 Jan 21;11:402. doi: 10.1038/s41467-019-13960-2 (PMC6972792; doi:10.1038/s41467-019-13960-2)
Supplement: Supplementary file 7 — Source data [file 41467_2019_13960_MOESM7_ESM.zip › Source_Data/FigureS2/FigureS2B_MotifScanOutput/KmeansCluster4/homerResults/motif13.similar.html]

motif13

## Information for motif13

G
A
C
T
T
G
A
C
A
G
C
T
A
G
T
C
T
C
G
A
G
A
C
T
T
C
A
G
C
T
G
A
C
T
A
G
G
T
C
A
C
G
T
A
A
G
T
C
C
G
A
T
  
Reverse Opposite:  

C
G
T
A
T
C
A
G
G
C
A
T
A
C
G
T
G
A
T
C
A
G
C
T
A
G
T
C
C
T
G
A
A
G
C
T
T
C
A
G
T
C
G
A
A
C
T
G
C
G
T
A
  

|  |  |
| --- | --- |
| p-value: | 1e-66 |
| log p-value: | -1.535e+02 |
| Information Content per bp: | 1.695 |
| Number of Target Sequences with motif | 178.0 |
| Percentage of Target Sequences with motif | 3.94% |
| Number of Background Sequences with motif | 253.5 |
| Percentage of Background Sequences with motif | 0.61% |
| Average Position of motif in Targets | 104.2 +/- 43.1bp |
| Average Position of motif in Background | 100.7 +/- 71.5bp |
| Strand Bias (log2 ratio + to - strand density) | 0.0 |
| Multiplicity (# of sites on avg that occur together) | 1.21 |
| Motif File: | file (matrix) reverse opposite |

### Similar de novo motifs found

|  |  |  |  |  |  |  |  |
| --- | --- | --- | --- | --- | --- | --- | --- |
| Rank | Match Score | Redundant Motif | P-value | log P-value | % of Targets | % of Background | Motif file |
| 1 | 0.974 | A T G C G C T A T A C G G C A T A G C T G A T C A G C T A G T C T C G A A G C T C T A G T C G A C A T G C G T A | 1e-63 | -145.349150 | 3.92% | 0.64% | motif file (matrix) |
| 2 | 0.956 | A C G T T G A C A G C T A G T C T C G A G A C T C T A G C T G A T C A G C T G A C G T A G A T C | 1e-58 | -135.835427 | 3.65% | 0.60% | motif file (matrix) |
| 3 | 0.894 | A G C T A G T C C T G A A G C T C T A G G T C A C T A G C T G A C G T A A G T C C G A T | 1e-55 | -127.988391 | 3.39% | 0.54% | motif file (matrix) |
| 4 | 0.842 | A C T G G C T A C G A T T G A C A G C T G A T C C T G A A G C T C T A G C G T A | 1e-45 | -105.896133 | 3.70% | 0.82% | motif file (matrix) |
